# Supplementary material for: Anti-Inflammatory Effects of Low-Dose Glucocorticoids Compensate for Their Detrimental Effects on Bone Mineral Density in Patients with Rheumatoid Arthritis
Source: J Clin Med. 2021 Jun 30;10(13):2944. doi: 10.3390/jcm10132944 (PMC8268831; doi:10.3390/jcm10132944)
Supplement: Supplementary file 1 [file jcm-10-02944-s001.zip › jcm-1284566-supplementary.pdf]

Table S1. Baseline demographic and clinical characteristics of patients with RA after propensity score matching.

Variable	GC (N = 500)	No-GC (N = 100)	*p* Value
Age, mean (years)	62 ± 10.4	61 ± 8.41	0.48
Sex			0.803
Women, N. (%)	475 (95)	96 (96)	
Menopause, N. (%)	408 (86.1)	85 (85)	0.519
BMI, mean	23.3 ± 3.7	23.7 ± 3.33	0.285
Smoking, N. (%)	22 (4.4)	9 (9)	0.058
Alcohol, N. (%)	60 (12)	11 (11)	0.777
RF positivity, N. (%)	303 (60.6)	51 (51)	0.075
Anti-CCP Ab positivity, N. (%)	149 (38.8)	38 (41.3)	0.659
Disease duration (months)	114.7 ± 76.6	100.2 ± 58.8	0.44
DAS28-ESR	2.82 ± 1.1	2.59 ± 0.78	0.276
DAS28-CRP	2.2 ± 0.9	1.96 ± 0.59	0.113
Bone erosion, N. (%)	178 (35.7)	21 (21)	0.005*
Medication			
Glucocorticoids			
Cumulative dose before BMD measurements (g)	7.33 ± 6.81	5.99 ± 5.29	0.058
Mean dose between BMD measurements (mg/day)	2.3 ± 1.71	NA	NA
NSAIDs, N. (%)	316 (63.2)	61 (61)	0.546
Methotrexate, N. (%)	232 (46.4)	39 (39)	0.175
Hydroxychloroquine, N. (%)	336 (67.2)	70 (70)	0.585
Sulfasalazine, N. (%)	36 (7.2)	9 (9)	0.533
Tacrolimus/cyclosporin, N. (%)	97 (19.4)	13 (13)	0.131
Leflunomide, N. (%)	80 (16)	7 (7)	0.02*
Anti-TNF agents, N. (%)	9 (1.8)	1 (1)	> 0.999
Adalimumab, N. (%)	3 (0.6)	0 (0)	> 0.999
Etanercept, N. (%)	1 (0.2)	1 (1)	0.306
Golimumab, N. (%)	2 (0.4)	0 (0)	> 0.999
Infliximab, N. (%)	3 (0.6)	0 (0)	> 0.999
Abatacept, N. (%)	3 (0.6)	0 (0)	> 0.999
Tocilizumab, N. (%)	10 (2)	0 (0)	0.383
Rituximab, N. (%)	3 (0.6)	0 (0)	> 0.999
JAK inhibitors, N. (%)	8 (1.6)	0 (0)	0.364

RA: rheumatoid arthritis, GC: glucocorticoid, BMI: body mass index, RF: rheumatoid factor, Anti-CCP Ab: anti-citrullinated protein antibody, DAS: disease activity score, ESR: erythrocyte sedimentation rate, CRP: C-reactive protein, 25(OH) D: 25-hydroxyvitamin D, BMD: bone mineral density, NSAIDs: non-steroidal anti-inflammatory drugs, TNF: tumor necrosis factor, JAK: Janus kinase, NA: not applicable. **p* < 0.05

Table S2. Annual change in BMD and new-onset osteoporosis rates between the GC users and the control group after propensity score matching.

Variable	GC (N = 500)	No-GC (N = 100)	*p* Value
Annual mean value of the absolute difference between baseline and follow-up BMD (g/cm^2)			
Lumbar spine	0.013 ± 0.043	0.01 ± 0.04	0.683
Femoral neck	0.002 ± 0.032	−0.001 ± 0.018	0.564
Total hip	0.001 ± 0.029	−0.001 ± 0.02	0.392
Annual % change in BMD (g/cm^2)			
Lumbar spine	1.6 ± 4.7	1.26 ± 4.44	0.693
Femoral neck	−0.45 ± 4.44	−0.53 ± 2.62	0.51
Total hip	−0.42 ± 3.5	−0.32 ± 1.8	0.328
New-onset osteoporosis, N. (%)	21 (4.2)	6 (6)	0.494

BMD: bone densitometry, GC: glucocorticoid.

Table S3. Multiple linear regression analysis of risk factors associated with annualized BMD change after propensity score matching.

Variable	Lumbar Spine		Femoral Neck		Total Hip	
	β	*p* Value	β	*p* Value	β	*p* Value
Univariate						
Age	0.048	0.012*	0.005	0.783	0.001	0.923

Menopause	1.584	0.005*	−0.544	0.301	0.006	0.988
BMI	0.019	0.721	−0.009	0.858	−0.063	0.092
Smoking	0.793	0.372	−0.21	0.789	0.288	0.639
Alcohol	−0.052	0.931	−0.205	0.7	0.039	0.926
RF positivity	−0.34	0.385	−0.079	0.822	0.061	0.823
Anti-CCP Ab positivity	−0.081	0.856	0.398	0.34	0.148	0.614
Disease duration	0.003	0.219	−0.001	0.881	0.002	0.365
Baseline DAS28-ESR	0.696	0.003*	0.626	0.012*	0.418	0.022*
Baseline DAS28-CRP	0.406	0.162	0.289	0.346	0.142	0.524
ΔDAS28-ESR	−1.783	< 0.001*	−0.987	< 0.001*	−1.058	<0.001*
ΔDAS28-CRP	−1.964	< 0.001*	−1.051	< 0.001*	−0.87	<0.001*
Bone erosion	1.024	0.012	0.363	0.323	0.495	0.084
Cumulative GC dose	0.093	0.001*	0.038	0.15	0.071	0.001*
Calcium intake	−0.64	0.11	0.272	0.449	−0.092	0.745
Vitamin D intake	1.186	0.046*	1.069	0.045*	0.805	0.053
PPI use	0.211	0.785	0.113	0.43	0.841	0.761
Multivariate						
Age	0.011	0.698	0.004	0.863	−0.003	0.874
Menopause	−0.157	0.84	−0.448	0.547	−0.238	0.659
Baseline DAS28-ESR	−0.464	0.099	0.063	0.835	−0.334	0.117
ΔDAS28-ESR	−1.426	0.009*	−0.665	0.259	−1.904	<0.001*
ΔDAS28-CRP	−0.628	0.296	−0.265	0.69	0.957	0.042
Bone erosion	0.983	0.058	0.421	0.443	0.408	0.308
Cumulative GC dose	−0.024	0.5	0.051	0.172	0.026	0.347
Vitamin D intake	−0.335	0.633	0.636	0.411	0.238	0.66

BMD: bone mineral density, BMI: body mass index, RF: rheumatoid factor, Anti-CCP Ab: anti-citrullinated protein antibody, ESR: erythrocyte sedimentation rate, CRP: C-reactive protein, DAS: disease activity score, GC: glucocorticoid, PPI: proton pump inhibitor, NA: not applicable; ΔDAS28-ESR is DAS28-ESR at the follow-up point minus baseline DAS28-ESR. ΔDAS28-CRP is DAS28-CRP at the follow-up point minus baseline DAS28-CRP. *$p < 0.05$
